# Cytotoxicity and Bonding Property of Bioinspired Nacre-like Ceramic-Polymer Composites

**DOI:** 10.3389/fbioe.2022.913899

**Published:** 2022-05-23

**Authors:** Hui Sun, Kefeng Gao, Zhe Yi, Chengwei Han, Zengqian Liu, Qiang Wang, Qing Zhou, Zhefeng Zhang

**Affiliations:** ^1^ Liaoning Provincial Key Laboratory of Oral Diseases, School and Hospital of Stomatology, China Medical University, Shenyang, China; ^2^ Shi-Changxu Innovation Center for Advanced Materials, Institute of Metal Research, Chinese Academy of Sciences, Shenyang, China; ^3^ Liaoning Upcera Co., Ltd, Benxi, China; ^4^ School of Materials Science and Engineering, University of Science and Technology of China, Hefei, China

**Keywords:** dental materials, ceramic-polymer composite, zirconia, cytotoxicity, bonding strength

## Abstract

For clinical applications, non-cytotoxicity and good bonding property of dental restorative materials are the most essential and important. The aim of this study was to evaluate the potential for clinical applications of two novel bioinspired nacre-like ceramic (yttria-stabilized zirconia)-polymer (polymethyl methacrylate) composites in terms of the cytotoxicity and bonding property. The relative growth rates (24 h) of the Lamellar and Brick-and-mortar composites measured by CCK8 were 102.93%±0.04 and 98.91%±0.03, respectively. According to the results of cytotoxicity and proliferation experiments, the two composites were not cytotoxic to human periodontal ligament fibroblasts (HPDLFs) *in vitro*. Both composites exhibited improved bonding strength as compared to the Control group (Vita In-Ceram YZ). As the polymer content in the composite material increases, its bonding strength also increases, which enhances the application potential of the material in the field of dental restoration. Meanwhile, by controlling the direction of loading force in the shear test, the effect of microstructure on the bonding strength of anisotropic composites was studied. After sandblasted, the bonding strengths of the Lamellar group in the longitudinal and transverse shear directions were 17.56±1.56 MPa and 18.67±1.92 MPa, respectively, while of the Brick-and-mortar group were 16.36±1.30 MPa and 16.99±1.67 MPa, respectively. The results showed that the loading direction had no significant effect on the bonding strength of the composites.

## 1 Introduction

With the development of medical technology and material science, as well as the increasing requirements for the repair of tooth defects and missing, the research and development of dental materials become one of the hot research topics. Among the modern dental restorative materials of ceramics, zirconia materials are widely favored by clinicians and patients owing to their high strength, good biocompatibility, low radioactivity and good optical properties (Chen and Pan, 2019; Wu et al., 2020; Abo-Elmagd et al., 2021). However, the brittleness of zirconia may induce cracking and ultimately lead to the failure of repair. It was found that the tetragonal phase of zirconia was retained in a metastable state at room temperature by adding oxides such as magnesium oxide (MgO), yttria (Y_2_O_3_), cerium oxide (CeO_2_) and calcium oxide (CaO), enabling transformation-induced toughening to occur (Hannink et al., 2000; Al-Amleh et al., 2010; Grech and Antunes, 2019). Among them, yttria addition is the most commonly used for inducing toughening, which was reported to be effective to inhibit crack propagation (Hannink et al., 2000; Kohorst et al., 2011; Juntavee and Sirisathit, 2018; Fonseca et al., 2019). Currently, 3 mol% yttria-stabilized tetragonal zirconia polycrystalline (3Y-TZP) is a commonly used zirconia material in dentistry; but its hardness is approximately 4 and 24 times, respectively, higher than that of human enamel and dentin (Craig and Peyton, 1958; Xu et al., 1998; Kim et al., 2013). Its high hardness is a burden on the remaining root and can cause excessive wear on the opposing teeth during daily use. Therefore, in recent years, some zirconia composites with hardness close to that of natural teeth have been developed, such as 3M Lava™ Ultimate and UPCERA Hyramic (Sehgal et al., 2016; Zhang et al., 2016). Their main solution was to mix varying proportions of polymer phase into the zirconia feedstock. Adding a softer polymer component to zirconia is an effective way to lower its hardness.

In our previous study, we developed two bioinspired nacre-like ceramic-polymer composites composed of differing contents of yttria-stabilized zirconia and polymethyl methacrylate (PMMA) (Tan et al., 2019). The composites were prepared by infiltrating zirconia scaffolds made by freeze-casting with varying proportions of polymer phase. By modulating the micro-scale to nano-scale characteristics of the lamellar structure and brick-and-mortar structure, the Young’s modulus and hardness of the composite were made essentially equal to those of the dentin and enamel in human teeth. Its unique microstructure gives it excellent mechanical properties, including elasticity and hardness that match human teeth, outstanding damage tolerance, and high fatigue resistance (Tan et al., 2019; Tan et al., 2021). This particular combination of properties makes composites attractive for dental applications.

Non-cytotoxicity and good biocompatibility of new biomaterials are important prerequisites for their practical applications. Therefore, biological evaluation of a new material is necessary. There are many kinds of biological evaluation methods, which can be divided into three basic tests: cell test *in vitro*, animal test and clinical application test. Among them, the cytotoxicity evaluation of materials *in vitro* is the most basic. Zirconia has excellent biocompatibility. However, it was reported that the polymer materials might have biological toxicity, especially in the non-fully cured state (Ulker et al., 2009). Since the composites were added with different contents of polymer, the biosafety of the composites should be evaluated before clinical applications.

Bonding properties of the Zirconia materials with resin base or human dentin is another concern when applied in clinics. It is difficult for zirconia ceramics to obtain a stable bonding strength, which in turn affects its repair effect. How to improve the bonding quality of zirconia ceramics is one of the difficulties in the research of this kind of dental materials. Many scholars did a lot of researches to improve the bonding strength of zirconia materials, mainly focusing on the improvement by surface treatment. It mainly includes sandblasting, grinding with bur, acid etching, laser irradiation, tribochemical silica coating, and zirconia primers, etc. (Han et al., 2013; Usumez et al., 2013; Shin et al., 2014; Kirmali et al., 2015; Tokar et al., 2019). These methods increase the mechanical retention or chemical bonding of zirconia ceramic restorations by roughening the zirconia surface or increasing the silicon content, thereby increasing the bonding strength and its durability. However, these techniques are not widely used in clinical practice because of high cost and limited stability. Unfortunately, the existing sandblasting technology or grinding with bur cannot effectively roughen the extremely hard zirconia surfaces. As the hardness of the composite diminishes with the addition of polymer phase, sandblasting is likely to increase surface roughness of the composites, which will increase the micromechanical interlocking force. And the resin-based adhesive is recommended in clinic, so the chemical adhesion of the composite may be improved with the addition of polymer. Furthermore, through the unique processing technology, the composite has a clear anisotropy in nacre-like lamellar and brick-and-mortar architectures. Therefore, in the current study, we thoroughly evaluated the bonding performance of the composites, meanwhile examined the influence of loading direction with respect to the microstructure on the bonding strength.

As for a dental restorative material, durable and stable bonding is the key to ensure the long-term success of the restoration. The purpose of this study was to provide the experimental basis for better and more systematic evaluation of the potential of the novel zirconia-polymer composites for dental applications.

## 2 Materials and Methods

### 2.1 Material Preparation

**FIGURE 1:**
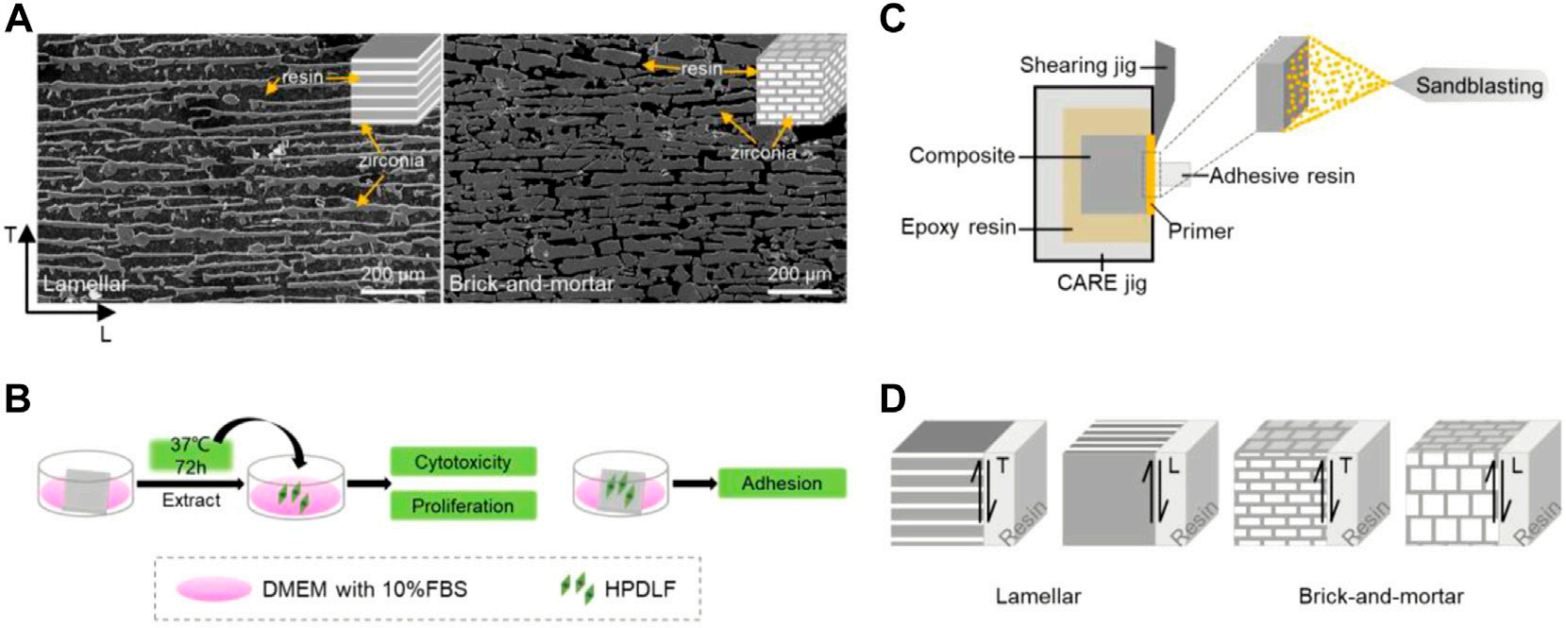
Flow diagram of this study. **(A)** Typical SEM images of the two composites; **(B)** simple flow charts for cell experiments; **(C)** model diagram of shear bonding strength test; **(D)** model diagram of experimental materials and shear direction (SEM image: dark black is resin, bright white is zirconia; model diagram: dark gray is resin, white is zirconia.)

The composite samples were prepared with 3Y-TZP suspensions and by infiltrating the freeze cast ceramic scaffolds with PMMA. More details were reported in our previous study (Tan et al., 2019). In the preliminary studies, the effects of different proportions of PMMA polymer on the mechanical properties of the composites were compared. Ultimately, two kinds of composites were selected for the following study. The two kinds of composites were named as the Lamellar and Brick-and-mortar ceramic-polymer composites (hereafter referred to as the Lamellar group and Brick-and-mortar group) respectively. The Lamellar group contains about 77 vol% polymer phase (lamellar structure). The hardness and Young’s modulus of the Lamellar group is close to those of human dentin. The Brick-and-mortar group contains approximately 20 vol% polymer phase (brick-and-mortar structure). The hardness and Young’s modulus of the Brick-and-mortar group are close to those of human enamel. Figure 1A shows typical scanning electron microscope images of the two composites with unique microstructures. Figures 1B–D illustrates the flow diagram of this study. The two groups of materials were processed into the dimension of 10 mm × 10 mm × 2 mm plate samples for biocompatibility test and 5 mm × 5 mm × 5 mm cube samples for shear test by the wire-cutting technology. All samples were sequentially polished gradually with SiC paper from 800 to 2000 grit with water-cooling. Then, all samples were ultrasonically cleaned in distilled water and absolute ethanol with an ultrasonic bath for 10 min, respectively, and dried with oil-free air spray. Finally, the samples for biocompatibility test were sterilized by autoclaving.

### 2.2 *In vitro* Cell Biocompatibility

#### 2.2.1 Extract Preparation

The Lamellar group and Brick-and-mortar group samples were immersed in Dulbecco’s modified Eagle’s medium (DMEM) containing 10% fetal bovine serum (FBS) for 72 h at 37°C in a humidified incubator with 5% carbon dioxide (CO_2_). According to ISO 10993 Part 12, the immersion ratio was 1.25 cm^2^/mL. The extracts were filtered and collected in a sterile manner. The extracts were prepared for evaluating the cell proliferation and cytotoxicity of the two ceramic-polymer composites.

Before and after extraction, four samples from each group were weighed using an analytical balance (XS105 Dual Range, Mettler Toledo, Switzerland) with precision of ±0.01 mg. Each sample was tested by three times to obtain an average value. It was utilized to simply assess the dissolving properties of the two ceramic-polymer composites.

#### 2.2.2 Cell Culture

Human periodontal ligament fibroblasts (HPDLFs) were cultured in DMEM medium with 10% FBS, and maintained in a humidified incubator with 5% CO_2_ at 37°C. When the monolayer grew to subconfluence, the cells were subcultured by 0.25% trypsinization (Sigma Chemical Co., St. Louis, MO). HPDLFs were used to test the cell proliferation, cytotoxicity and adhesion ability of the Lamellar group and Brick-and-mortar group samples.

#### 2.2.3 Cell Proliferation and Cytotoxicity Test

The CCK-8 cell viability assay (United States Everbright Inc, Silicon Valley, United States) was used to evaluate the cytotoxicity of the Lamellar group and Brick-and-mortar group samples. HPDLFs were incubated in 96-well cell culture plates (Corning, NY) at a density of 1×10^3^ cells/well. Following 24 h adhesive culture at 37°C the medium was removed and replaced with 100 μL of extracts, whereas the control groups were replaced by normal culture medium, with six biological replicates per group. The 96-well cell culture plates were incubated in a humidified atmosphere with 5% CO_2_ at 37°C for 24 h, 48 h, 72 h, and 120 h, respectively. Then after adding 100 μL of DMEM with 10% CCK-8 to each well, the 96-well cell culture plates were incubated at 37°C for 2 h. The spectrophotometric absorbance of the samples was measured at 450 nm (500 nm) with a microplate reader (Infinite M200, Tecan, Austria). All tests were repeated three times. Statistical analysis was performed with SPSS 22.0 software. Differences between groups were analyzed using one-way ANOVA followed by Tukey’s test.

#### 2.2.4 Cell Adhesion Assay

HPDLFs were seeded onto the Lamellar group and Brick-and-mortar group samples in 24-well plates at a density of 1×10^4^ cells/well. After removing the culture media, samples were fixed with 2.5% glutaraldehyde solution for 4 h at 4°C after 6 h and 24 h incubation respectively. All the samples were washed 3 times with phosphate buffer solution (PBS, pH = 7.4), and subsequently dehydrated in a graded ethanol series (30, 50, 75, 95, and 100 vol%) for 10 min each. The morphologic characteristics of the cells cultured onto the samples’ surfaces were observed by scanning electron microscopy (SEM, Hitachi S-3400N, Japan).

HPDLFs were seeded onto the Lamellar group and Brick-and-mortar group samples in 24-well plates at a density of 1×10^4^ cells/well for 4 h, 24 h and 72 h, respectively. After that, the samples were washed with phosphate buffer solution 3 times, followed by fixing with 4% paraformaldehyde for 20 min. The cytoskeleton and cell nuclei were stained by rhodamine-phalloidin and DAPI in dark respectively and observed by fluorescence microscopy (ZEISS, Germany).

### 2.3 Shear Bonding Strength (SBS) Test Along Different Directions

#### 2.3.1 Surface Treatment

Zirconia ceramics (Vita In-Ceram YZ, Vita Zahnfabrik, Bad Säckingen, Germany) were selected as the control group and also processed into 5 mm × 5 mm × 5 mm cubes. All the samples for bonding strength tests were embedded in epoxy resin and ensured that in the Lamellar group and Brick-and-mortar group the oriented faces were exposed. All the samples were successively polished with SiC paper from 800 to 2000 grit gradually with water-cooling. And then, all the samples were ultrasonically cleaned in distilled water and absolute ethanol with an ultrasonic bath for 10 min, respectively, and dried with oil-free air spray. Next, half of the Lamellar group, Brick-and-mortar group and the Control group samples were randomly selected for sandblasting. The exposed oriented surfaces were sandblasted, using a sandblasting machine (Basic quattro IS, Renfert, Germany) for 10 s with 50 µm silica modified Al_2_O_3_ particles with 2.5 bar pressure from 10 mm distance. After sandblasted, the samples were completely rinsed with water spray for 30 s to clean the residual sand particles from the surface, and dried.

#### 2.3.2 Surface Characterization

The surface roughness values (Ra) of the Lamellar group and Brick-and-mortar group samples before and after sandblasted were evaluated using a 3D white-light interfering profilometer (MicroXAM 3D, ADE Corp, United States). The average roughness of each sample was calculated by examining three random samples in each group.

To characterize the wettability of the Lamellar group, Brick-and-mortar group and Control group samples before and after sandblasted, the contact angle (CA) of samples with water was measured by the wettability measuring instrument (OCA 25, DataPhysics Instruments Gmbh, Germany) through SCA20 software. Each group of samples needed to be measured on the left and right sides and repeated five times to reduce the error.

#### 2.3.3 Preparation of Standard Specimens for Shear Bonding Strength Test

The specimen preparation method was the same for all groups. Specimens were chemically adhered using a bonding primer containing 10-methacryloyloxydecyl dihydrogen phosphate (MDP) (Z-Prime Plus, Bisco, Schaumburg, IL, United States) and a dual-cured adhesive resin cement (RelyX U200, 3M ESPE, MN) following the manufacturer’s instructions. A piece of double-coated tape with one circular holes (2.2 mm in diameter) was positioned on the sample surface to define the bonding area. Then the plastic cylinder (2.2 mm in inside diameter and about 2 mm in depth) was fixed to the surface of the sample so that it had the same center with the hole. A thin layer of Z-primer Plus was applied evenly, followed by the RelyX U200 filling the model. After that, light-polymerization was performed for 10 s in each of the four directions over a 1 mm range, using a light-polymerizing device (Astralis 3, Ivoclar Vivadent, Liechtenstein) with an output power of 600 mW/cm^2^. After removing the plastic cylinder, an additional 20 s polymerization was performed; and then the tape was removed from the surface of specimen. All bonded specimens were stored in distilled water at 37°C for 24 h before the SBS test.

Compared with the Control group, the Lamellar group and the Brick-and-mortar group showed anisotropic orientations on the exposed bonding surfaces. So, the transverse (T) and longitudinal (L) shear forces were used for the Lamellar group and the Brick-and-mortar group specimens in the SBS test, whereas the shear direction was not controlled in the Control group. There were a total of 10 experimental groups. Each group consisted of 15 specimens, of which 12 were used for the shear test and three for observation of the bonding interface.

#### 2.3.4 Shear Bonding Strength Measurement

Subsequently, SBS was measured at a 0.5 mm/min crosshead speed using a universal testing machine (CARE M-3000, Kell measurement and control Co., Ltd, China) until failure. The SBS value was calculated by dividing the peak load at failure by the bonded surface area as follows:

$$\text{SBS}\left(\text{MPa}\right)=\text{load}\left(\text{N}\right)\text{/area}\left({\text{mm}}^{2}\right)$$ (1)

#### 2.3.5 Failure Mode Analysis

After the SBS test, all the samples were observed at a magnification of 29× using a dental operating microscope (OMS2355, Zumax Medical Co., Ltd, Suzhou, China) to identify the failure mode. Failure modes were classified as the adhesive failure at the sample-resin cement interface, cohesive failure within resin cement, and mixed failure where adhesive and cohesive failures occur at the same time. The failure morphologies were characterized by SEM.

#### 2.3.6 Observation of the Bonding Interface

The longitudinal section along the diameter of the remaining sandblasted Lamellar group and the Brick-and-mortar group specimens was cut with a slow speed cutting machine under the cooling of flowing water. After polished with SiC paper from 800 to 2000 grit gradually with water-cooling, ultrasonic washed with distilled water for 2 min, critical point dried, and coated with gold, the bonding interface was observed by SEM.

#### 2.3.7 Statistical Analysis

All statistical analyses were carried out using SPSS 22.0 software at a level of significance of α = 0.05. The parametric one-way analysis of variance (ANOVA) test was used to check differences in mean scores between groups. Pairwise comparison was done using Tukey’s honestly significant difference (HSD) post hoc test.

## 3 Results

### 3.1 *In vitro* Cell Biocompatibility

#### 3.1.1 Cell Proliferation and Cytotoxicity

The weight changes of the Lamellar and Brick-and-mortar group samples before and after the extract preparation were shown in Table 1. It showed that there was almost no weight change.

**TABLE 1:** Weight changes before and after sample extract preparation.

Samples	Lamellar (g)				Brick-And-mortar (g)			
	L1	L2	L3	L4	B1	B2	B3	B4
Before extract preparation	0.4469	0.4343	0.4445	0.4430	0.8083	0.8010	0.8054	0.8186
After extract preparation	0.4472	0.4347	0.4449	0.4434	0.8090	0.8018	0.8060	0.8191
∆m	-0.0003	−0.0004	−0.0004	−0.0004	−0.0007	−0.0007	−0.0006	−0.0005

**FIGURE 2:**
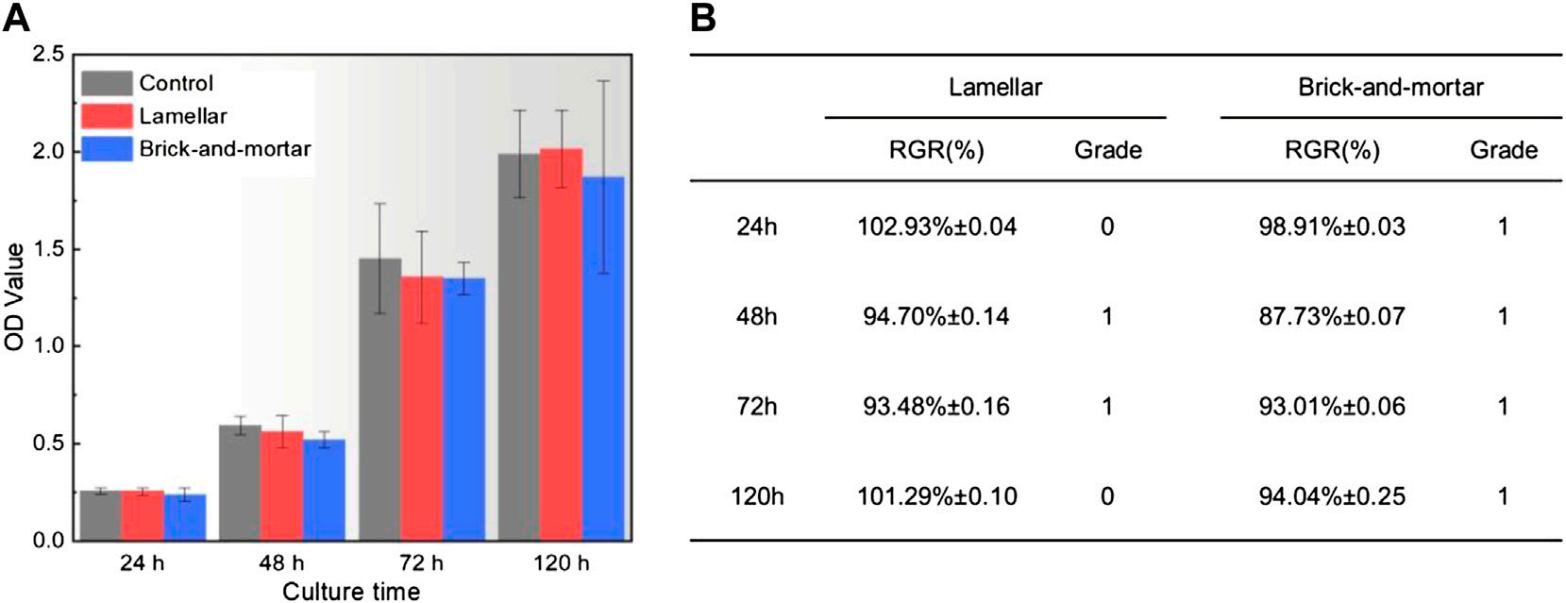
**(A)** Optical density of HPDLF cells in extraction medium of experimental materials measured by CCK8 test and **(B)** relative growth rate and cytotoxicity level at different detection period.

The optical densities (OD) values of HPDLF cells in different extracts were measured by the CCK8 test (Figure 2A). The results showed that the absorbance values gradually increased with the increase of the culture time. There were no significant differences among all the groups on 24 h, 48 h, 72 h, and 120 h (*p* > 0.05). In Figure 2B, the relative growth rate (RGR) of HPDLF cells in extracts of the Lamellar and Brick-and-mortar composites were assessed and calculated as follows:

$${\text{RGR}=\text{OD}}_{\text{e}}{\text{/OD}}_{\text{c}}\text{×}100\text{\%}\text{.}$$ (2)

OD_e_ is the average OD value of the experimental groups; and OD_c_ is the average OD value of the control group. The cell toxicity grade (CTG) was obtained according to the standard United States Pharmacopeia (Aminoroaya et al., 2020).

It was found that CTG of the bioinspired ceramic-polymer composites were in grade 1 or 0 which indicates no toxicity. Merely based on the OD values, the Lamellar composite displayed slightly better biocompatibility than the Brick-and-mortar composite.

#### 3.1.2 Cell Morphology and Adhesion

**FIGURE 3:**
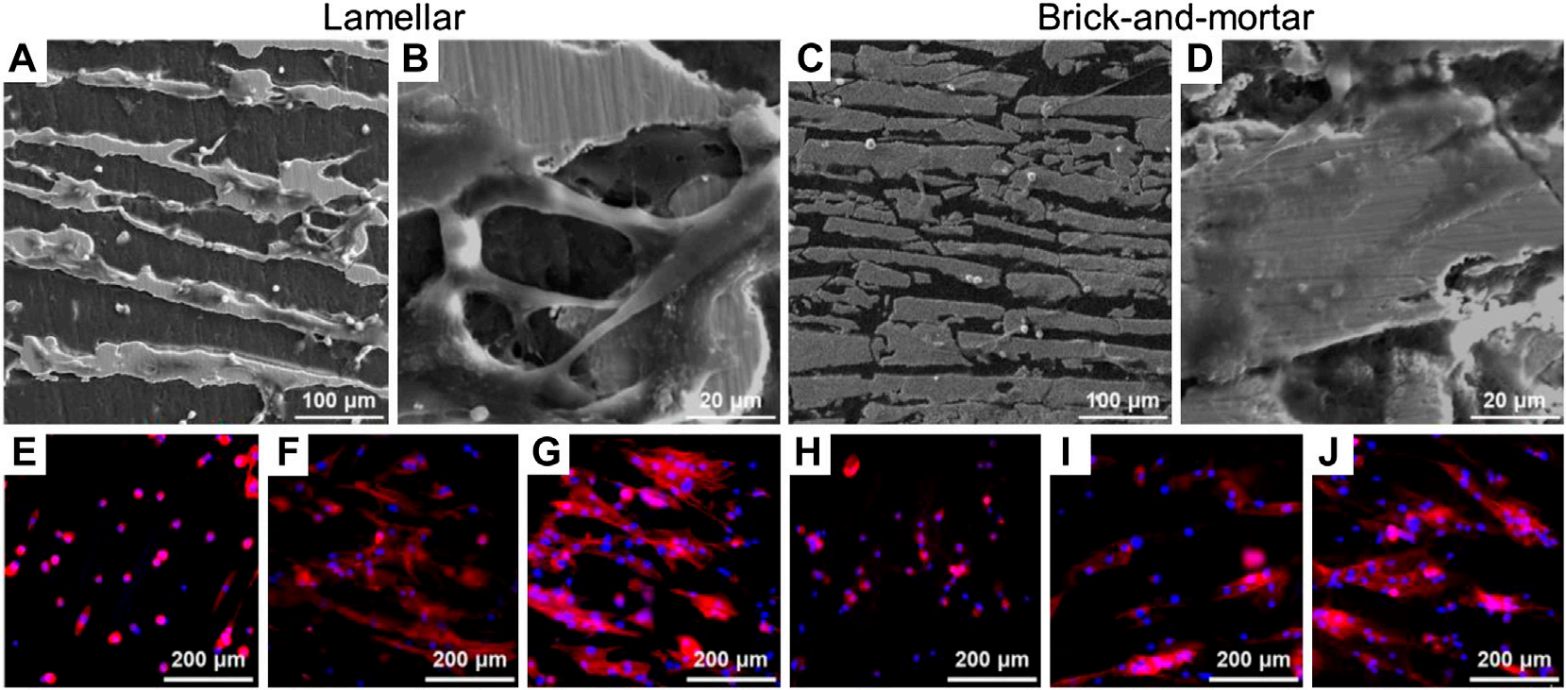
Adhesion of HPDLF cells on different samples. **(A–D)** Scanning electron microscopy morphologies of co-culture **(A,B)** Lamellar for 24 h, **(C,D)** Brick-and-mortar for 24 h **(E–J)** Cytoskeleton staining of the experimental samples of co-culture, which DAPI for nuclei (blue) and rhodamine-phalloidin for F-actin stress fibers (red) double stain: **(E)** Lamellar for 6 h, **(F)** Lamellar for 24 h, **(G)** Lamellar for 72 h, **(H)** Brick-and-mortar for 6 h, **(I)** Brick-and-mortar for 24 h, **(J)** Brick-and-mortar for 72 h.

The morphologies of HPDLF cells cultured on the Lamellar and Brick-and-mortar composites at different time were shown in Figure 3. After 6 h of culture, cells were attached onto all the samples. After 24 h of culture, cells spread well on all samples, exhibiting a flat morphology of predominantly fusiform. The number of cells increased significantly with the extension of culture time (Figure 3G and Figure 3J).

### 3.2 Shear Bonding Strength Test Along Different Directions

#### 3.2.1 Characterization of Material Properties Before and After Sandblasted

**FIGURE 4:**
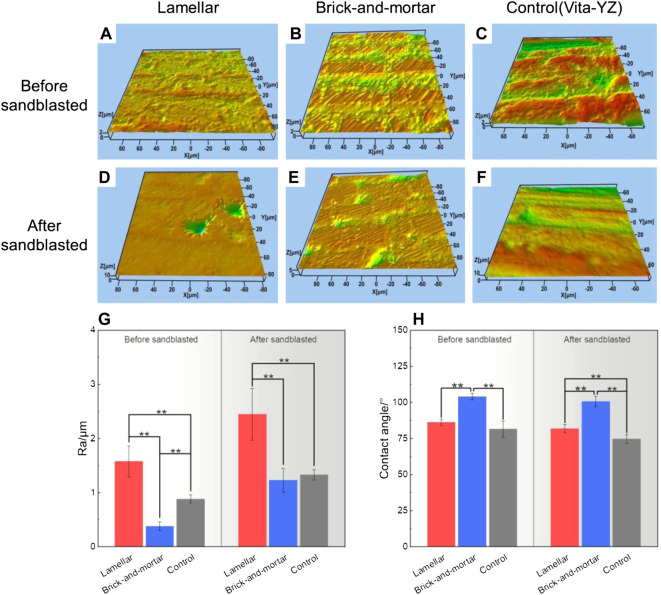
Surface three-dimensional morphology **(A–F)**, surface roughness **(G)** and contact angle **(H)** of all groups.

Figures 4A–F shows the representative three-dimensional surface morphology image of all materials before and after sandblasted. The results showed that the two composites still had anisotropic orientations after sandblasted. The surfaces of the blasted samples were dotted with uneven dents (Figure 4D and Figure 4E), which mainly occurred on the polymer phase. The surface roughness was shown in Figure 4G. The Ra values of the Lamellar and Brick-and-mortar composites after sandblasted were significantly larger than those before sandblasted with the differences statistically significant (*p < 0.05*).

The hydrophilicity measurement results were shown in Figure 4H. The Lamellar, Brick-and-mortar and Control groups before sandblasted had the contact angle (CA) of 86±2.03°, 104±2.37° and 82±5.58°, respectively. In comparison, the CA of samples after sandblasted was 82±2.72°, 101±3.45° and 75±3.02°, respectively. Although the differences for the Control group before and after sandblasted was statistically significant (*p* < 0.05), the CA values of Lamellar and Brick-and-mortar groups after sandblasted only exhibited a small decrease, with no significant significance (*p* > 0.05).

#### 3.2.2 Shear Bonding Strength

**FIGURE 5:**
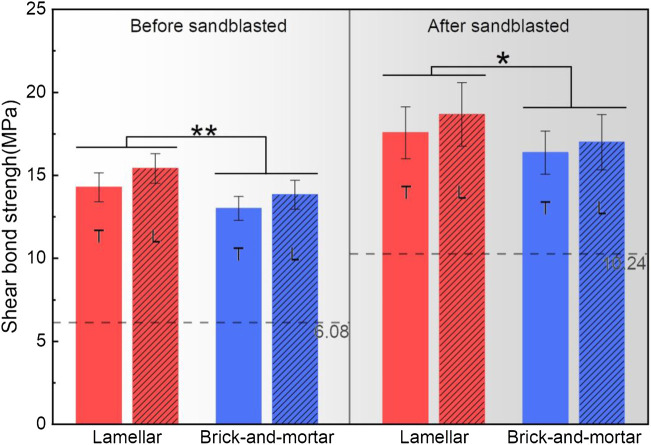
Graphical representation of mean shear bonding strength values (±SD) of all groups. The dashed line represents the bonding strength of the control group (Vita In-Ceram YZ) *: statistically significant differences (*n* = 12, *p < 0.05*).

The statistical comparisons of the SBS were shown in Figure 5. The SBS values before and after sandblasting treatment were comparable under the same shear direction and material type. The SBS values for the Lamellar and Brick-and-mortar groups after sandblasted were significantly higher than those before sandblasted (*p < 0.05*). However, there was no significant difference for the Control group.

The SBS values of different materials under the same surface treatment and the same shear direction were compared. Regardless of shear direction, the respective SBS values of the Lamellar and Brick-and-mortar groups were significantly higher than those of the Control group (*p < 0.05*). And, the SBS value of the Lamellar group was significantly higher than that of Brick-and-mortar group (*p < 0.05*).

The SBS values along different shear directions were also compared. In both Lamellar and Brick-and-mortar groups, there were no significant differences between longitudinal and transverse shear directions (*p > 0.05*).

#### 3.2.3 Failure Mode Analysis

**FIGURE 6:**
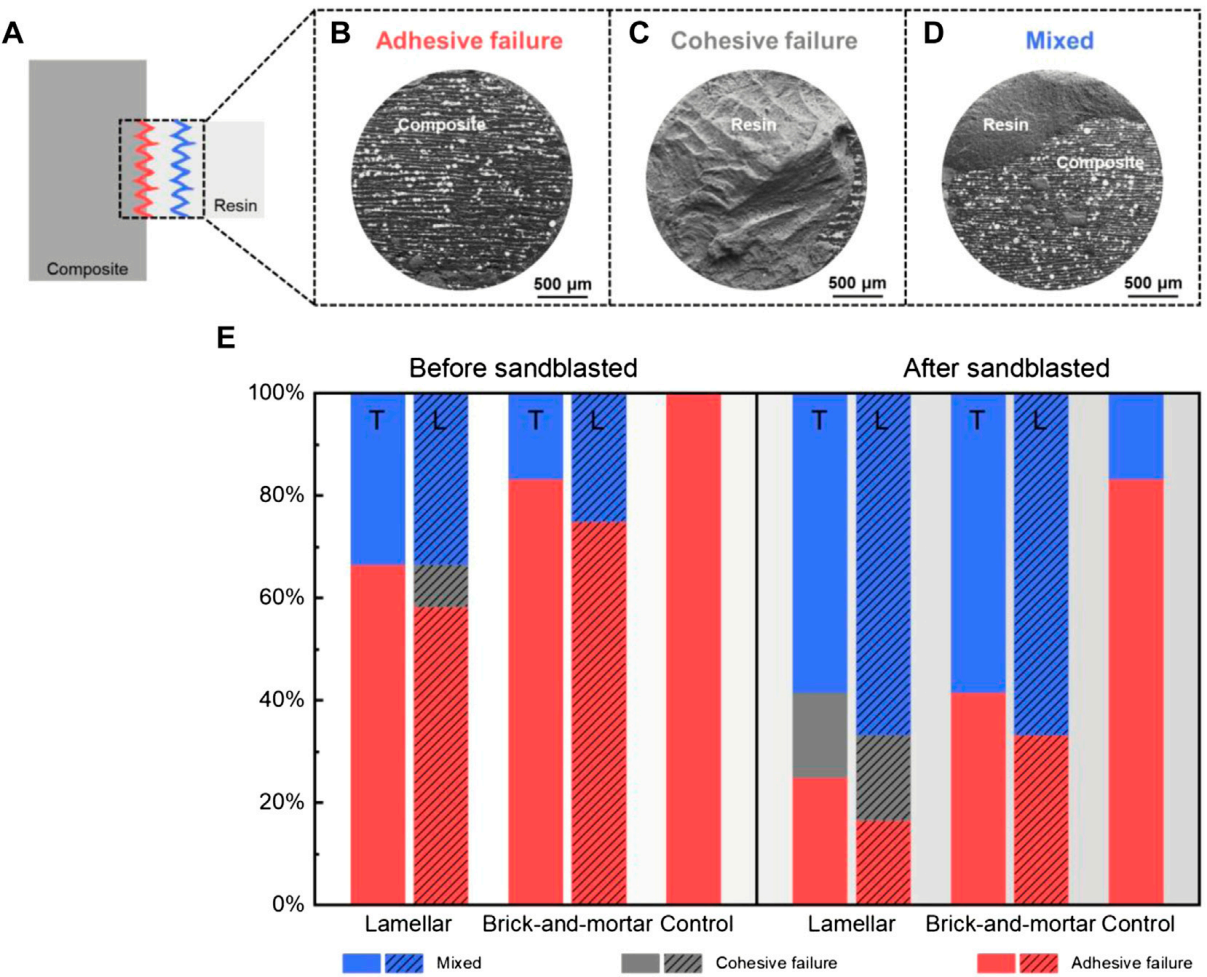
Taking the failure mode **(A)** of the Lamellar material as an example, there are three typical modes: **(B)** adhesive failure, **(C)** cohesive failure, **(D)** mixed mode. **(E)** Illustration and schematic diagram of the percentages of the failure modes after the shear bonding strength test of all groups.

Figures 6A–D show a schematic diagram of the various failure modes. The percentage of fracture surfaces with different failure modes for each group was shown in Figure 6E. By comparing the three material groups, there was no cohesive failure within resin cement in the Brick-and-mortar group and Control group. According to the surface treatment method, the failure mode of the specimens after sandblasted was mainly the mixed failure where adhesive failure and cohesive failure occur at the same time; whereas the specimens without sandblasting treatment mainly exhibited the adhesive failure at the zirconia-resin cement interface.

#### 3.2.4 Observation of Bonding Interface

**FIGURE 7:**
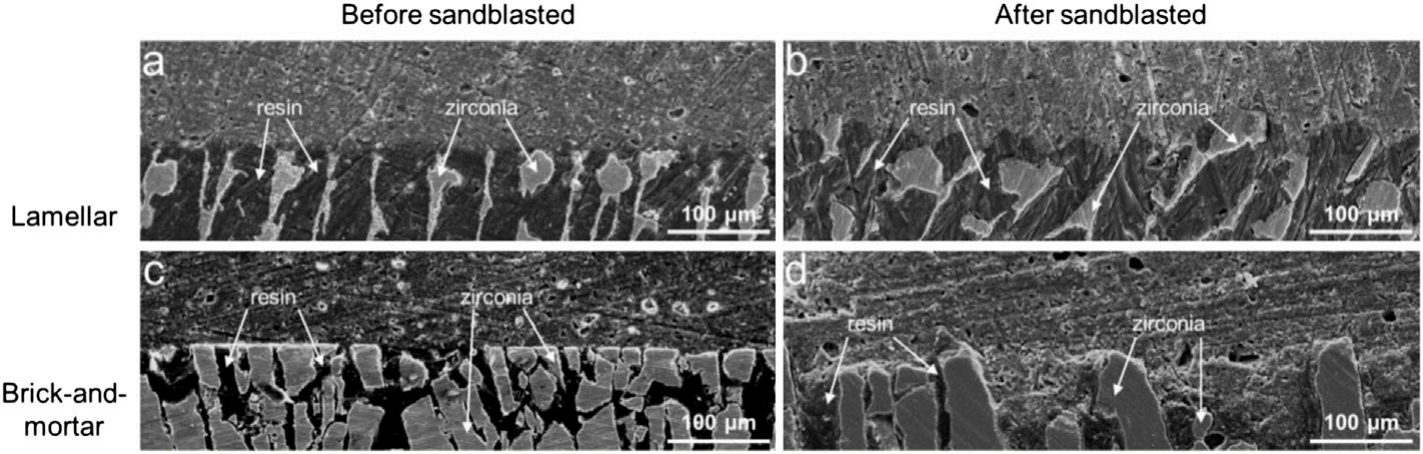
SEM images of the bonding interfaces with resin for the Lamellar and Brick-and-mortar groups before and after sandblasted.

The SEM images of the bonding interfaces of the Lamellar group and Brick-and-mortar group were shown in Figure 7. The overall bonding interface between composites and resin cement had a good tightness. Microcracks could only be seen in part of the ceramic phase. In addition, for the Lamellar group after sandblasted, more resin cement could be seen to protrude into the composite, forming the micro-mechanical interlocking interface.

## 4 Discussion

### 4.1 *In vitro* Cell Biocompatibility

Cytotoxicity is the primary consideration for the clinical applications of new biomaterials (Li et al., 2021). Several previous studies have demonstrated that zirconia is not cytotoxic (Christel et al., 1989; Piconi et al., 1998; Lohmann et al., 2002; Manicone et al., 2007; Shin et al., 2016). However, Shin et al. suggested that although pure zirconia was not cytotoxicity, the commercial zirconia block might have low toxicity according to the different cell lines (Shin et al., 2016). The clinical application of methacrylic acid polymer in oral cavity is also controversial. The acrylic resin such as PMMA in the oral mucosa is prone to allergic reactions, especially considering that its residual monomers even have potential cytotoxicity (Lee et al., 2020). But at present, polymer-based materials are still widely used in prosthodontics and orthodontics owing to their aesthetic properties (Leyva Del Rio et al., 2020). Therefore, we preliminarily evaluated the cytocompatibility of the materials by cell proliferation, cytotoxicity test and cell adhesion assay.

In the *in vitro* test of HPDLF cells, the Lamellar and Brick-and-mortar composites with different contents of polymer phase had no significant effects on the proliferation ability of fibroblasts compared with negative control group (normal cells) (Figure 2). All the zirconia-PMMA samples were non-cytotoxic. It indicated that the two composites had no negative effects on the cell proliferation and morphology in this study. This may be due to the good stability of the polymer-containing zirconia composites. The response of fibroblasts to the Lamellar and Brick-and-mortar composites was assessed by observing the morphologies of HPDLF cells cultured on the two composites in this study. HPDLF cells on the Brick-and-mortar surfaces were more easily attached and spread out better at 24 h than those in the Lamellar group.

This may be related to the hydrophilicity of the two materials (Figure 4H). Compared with the Brick-and-mortar group, the Lamellar group has smaller contact angle and better hydrophilicity, resulting in more adhesion and better extension of fibroblasts on its surface. We speculate that this may be due to the fact that the addition of more polymer phase (than the Brick-and-mortar one) will reduce the bioactivity of the composite. To sum up, according to the cytotoxicity classification, the two composites were considered to have no significant cell cytotoxicity.

In addition, in this experiment, 100% material extract was used to culture cells in order to study whether cytotoxicity was related to material dissolution and to discern the dose-dependent relationship between dissolution concentration and cytotoxicity. Some scholars detected the release of zirconium and yttrium ions in artificial saliva at 37°C with the amount of ion release increasing under acidic conditions (Green, 1983). Compared with zirconia and the major components of other dental ceramics, PMMA has a relatively higher solubility (Vojdani and Giti, 2015). Interestingly, there was no significant change in the weight of the two samples before and after extract preparation (Table 1). This may be due to the short extraction time or the preparation conditions of the extract which did not simulate the oral slightly acidic conditions. The cytotoxicity of the materials after aging at different times in simulated oral environment is worthy of further study.

### 4.2 Shear Bonding Strength Along Different Directions

At present, the preservation of restorations is overwhelmingly dependent on the strong bonding strength. Poor bonding may lead to loosening or dislodging of the dental restorations. Therefore, the bonding ability is very important and is the main factor required for the restoration to perform its function in long-term durations, especially for the silica-free zirconia ceramic restorations (Li et al., 2017). Although there is no consensus on a specific bonding procedure when treating zirconia restorations (Cavalcanti et al., 2009; Tanış et al., 2018), the method of using resin cements combined with preliminary zirconia surface treatment is highly recognized (Mizrahi, 2008; Yassaei et al., 2015). The present study evaluated the commonly used clinical bonding procedures of sandblasting treatment combined with resin-based adhesives containing 10-MDP to determine the bonding strength between the resin cement and the composite materials. The results showed that after sandblasted, the bonding strength of each group was significantly improved. Previous studies showed that this mechanical surface treatment improved the bonding strength by increasing the bonding surface area, surface roughness and wettability of the zirconia material surface (Tanış et al., 2015). Similar results were obtained in this study. After the surface of the composite material was sandblasted, its roughness and hydrophilicity increased (Figure 4G and Figure 4H). The results showed that the contact angle of all materials decreased after sandblasted. It indicated an increase in wettability, enabling the resin binder to flow into the composite surface (Ju et al., 2019; Alaqeel, 2020). Although it failed to achieve super-hydrophilicity, improving the surface wettability of the material can enhance the bonding strength between the repair material and the resin adhesive. The hydrophilicity and wettability of the methyl methacrylate resin may enable the two composites to have good wettability, which is beneficial to the bonding strength (Wang et al., 2021). The bonding strength of the Lamellar group was higher than that of the Brick-and-mortar group, probably due to the addition of more resin phase. The poor bonding strength of the Control group may be due to the lack of resin phase. However, the direct relationship between the bonding strength and contact angle cannot be determined because of the confounding of hydrophilicity, wettability, and surface energy (Degirmenci and Saridag, 2020). Ozcan et al. (Özcan et al., 2008) pointed out that MDP monomer could be directly bonded with metal oxides. The bonding strength mainly depends on the reaction between the hydroxyl groups in the MDP monomer and the hydroxyl groups on the zirconia surface. Nonetheless, Yassaei et al. (Yassaei et al., 2015) found that, without any surface preparation, only increasing the volume and flow of functional monomer cannot effectively improve zirconia adhesion. Similarly, several researchers confirmed that resin cement containing MDP could not achieve durable bonding with zirconia ceramics without surface treatment (Amaral et al., 2014; Samimi et al., 2015; Saade et al., 2020). Therefore, due to the superposition of the above two effects, the bonding strength of the composite materials in this experiment is much higher than that of the control group (pure zirconia material). The failure mode analysis showed that the cohesive failure within resin cement occurred only in the Lamellar group. For the two composite materials, the specimens without sandblasting treatment mainly exhibited the adhesive failure, while the frequency of the mixed failure increased in the specimens after sandblasted. It showed that the surface treatment method of sandblasting is effective to improve the bonding strength. However, in the Control group, regardless of sandblasted or not, most of the failure modes were the adhesive failure at the zirconia-resin cement interface, indicating that the bonding strength between the two composites and the adhesive resin cement was stronger than that of pure zirconia material. This may be due to the choice of a resinous adhesive, which may have some reaction with the resin phase of the composites. Table 2 summarized the bonding properties of different zirconia materials. Some literatures whose research models were consistent with or similar to this study were screened. However, due to the differences in sandblasting parameters, adhesives and experimental machines, the bonding strength varies greatly, ranging from about 4 to 35 MPa. The bonding strength of the two composites is also within this range. In the present study, the resin cements exhibited shear bonding strength values higher than the recommended minimum, which should not be lower than 10-12 MPa in oral clinical applications (Ab-Ghani et al., 2015).

**TABLE 2:** Experimental studies on resin bonded dental zirconia.

Material	Manufactured by	Adhesive	Mean Bonding Strength (MPa)	References
3Y-TZP	Nacera, Dortmund, Germany	SuperCem	4.23±0.84	El-Wassefy et al. (2021)
IPS e.max ZirCAD MO	Ivoclar Vivadent AG	OptiBond Solo Plus	9.9±2.6	Sadid-Zadeh et al. (2021)
Katana (Ultra translucent multilayered Y-PSZ)	Kuraray Noritake Dental Inc., Tokyo, Japan	Variolink LC	17.3±3.2	Noronha et al. (2020)
VITA In-Ceram YZ	Vita Zahnfabrik, Bad Säckingen, Germany	Clearfil bond	13.44±1.28	Zakavi et al. (2019)
VITA In-Ceram YZ	Vita Zahnfabrik, Bad Säckingen, Germany	Futrabond U	16.87±1.83	Zakavi et al. (2019)
ICE Zirkon	Zirkonzahn, Italy	Variolink	8.57±4.72	Çakırbay Tanış et al. (2019)
ICE Zirkon	Zirkonzahn, Italy	Panavia	35.17±9.99	Çakırbay Tanış et al. (2019)
D max Omega Dark	DMAX Co., Daegu, Korea	Permacem 2.0	21.24±8.97	Go et al. (2019)
D max Omega Dark	DMAX Co., Daegu, Korea	Clearfil SA Luting	12.76±10.35	Go et al. (2019)
VITA YZ	VITA YZ GmbH-Germany	Single bond universal (3M ESPE)	19.18±4.38	Akbarzadeh et al. (2018)
VITA YZ	VITA YZ GmbH-Germany	All bond universal (Bisco)	18.48±3.2	Akbarzadeh et al. (2018)
Prettau zircon	Zirkonzahn, Italy	AdperTM Single Bond 2 Adhesive	7.80±0.76	Han et al. (2013)

**FIGURE 8:**
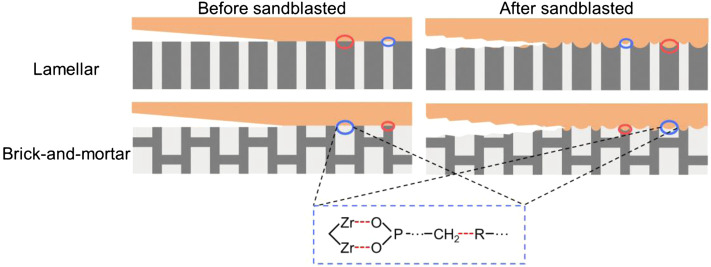
Possible bonding mechanism diagrams at the bonding interface of the Lamellar and Brick-and-mortar group.

In this study, the bonding strength of the lamellar composite containing 77vol% polymer phase was significantly higher than that of the brick-and-mortar one. The differences were statistically significant both before and after sandblasting treatment. A possible bonding mechanism of the bioinspired ceramic-polymer composites with Lamellar and Brick-and-mortar structures was shown in Figure 8. According to the current mechanical theory, the adhesive must penetrate into the voids inside the bonding surface as much as possible to remove the air bubbles on the interface so as to promote bonding (Li et al., 2017). Sandblasting could effectively amplify the penetration of the adhesives into the micro-pits and micro-hollows on the surface of zirconia, thereby increasing the bonding area and mechanical interlocking effect. In addition, the close combination of the bonding primer to the zirconia increased the intermolecular forces and the bonding strength. Furthermore, primers or resin cements containing functional monomers could increase the chemical bonding and wettability. The interface formed by the combination of physical and chemical bonding is more reliable and stable, which is likely to foster the longevity of bonded clinical restorations. Therefore, among the influencing factors of bonding strength, chemical bonding may play a leading role, resulting in better bonding properties of composites with high polymer content.

The effect of different shearing directions on the bonding strength of the composites (zirconia-polymer) was assessed. Although the bonding strengths of the two experimental materials under transverse shearing load are higher than those along longitudinal direction, the differences between them were not statistically significant. There are three possible reasons for this. The first and most important reason is that regardless of the shearing load, the effective area of bonding is the same where the chemical bonding of the polymer phase plays a leading role. Secondly, the penetration of the bonding resin monomers into the pits on the surface of the restorative material using sandblasting technology results in the bonding of resin adhesives to the restorative material (zirconia composite material). This process is also random along both directions and causes no obvious difference between different directions. The last possible reason is that the choice of the bonding strength test model is the microshear test where the effective bonding area may be too small to reflect the differences between loading directions (Otani et al., 2015). Of course, these are speculative reasons and further studies need to be conducted.

However, since the existence of different oral environments, such as bacteria, temperature and humidity, and pH level changes, the bonding strength of dental restorative materials may be significantly affected. Further related studies are currently in progress.

## 5 Conclusion

The following conclusions can be drawn from this study:

1) The bioinspired Lamellar and Brick-and-mortar composites have good biocompatibility and generally meet the standards for clinical use as dental restorations. The cytotoxicity test results (24 h) showed that the cell toxicity grade of the Lamellar and Brick-and-mortar composites were in grade 0 and grade 1, respectively. It indicated that the addition of polymer phase had little effect on the cytotoxicity of the composites. The early adhesion morphology of HPDLF cells on the materials was not changed.
2) The bonding performance of the Lamellar and Brick-and-mortar composites was better than that of zirconia ceramics. Shear bonding strength test showed that the sandblasting treatment helped to improve the bonding strength of the composites. In addition, the bonding strength of the Lamellar composite which has more polymer content (average 18.12±1.80 MPa after sandblasted) was higher than that for the Brick-and-mortar one (average 16.68±1.50 MPa after sandblasted). However, the anisotropy of microstructure of the two composites had no significant effect on the bonding strength.

## Data Availability

The original contributions presented in the study are included in the article/Supplementary Material, further inquiries can be directed to the corresponding authors.
